# Role of protective stoma after primary anastomosis for generalized peritonitis due to perforated diverticulitis—DIVERTI 2 (a prospective multicenter randomized trial): rationale and design (nct04604730)

**DOI:** 10.1186/s12893-022-01589-w

**Published:** 2022-05-16

**Authors:** Jean Pinson, Jean-Jacques Tuech, Mehdi Ouaissi, Murielle Mathonnet, François Mauvais, Estelle Houivet, Elie Lacroix, Julie Rondeaux, Charles Sabbagh, Valérie Bridoux

**Affiliations:** 1 Department of Digestive Surgery, Rouen University Hospital, Rouen, France; 2 Department of Digestive Surgery, Tours University Hospital, Chambray-lès-Tours, France; 3 Department of Digestive Surgery, Limoges University Hospital, Limoges, France; 4 Department of Digestive Surgery, Beauvais General Hospital, Beauvais, France; 5 Department of Biostatistics, Rouen University Hospital, Rouen, France; 6 Department of Digestive Surgery, Amiens University Hospital, Amiens, France; 7 Department of Digestive Surgery, Rouen University Hospital, 1 rue Germont, 76031 Rouen Cedex, France

**Keywords:** Ileostomy, Perforated diverticulitis, Hartmann’s procedure, Rectosigmoid resection, Peritonitis, Primary anastomosis, Quality of life

## Abstract

**Background:**

Traditionally, patients with peritonitis Hinchey III and IV due to perforated diverticulitis were treated with Hartmann’s procedure. In the past decade, resection and primary anastomosis have gained popularity over Hartmann’s procedure and recent guidelines recommend Hartmann’s procedure in two situations only: critically ill patients and in selected patients with multiple comorbidity (at high risk of complications).

The protective stoma (PS) is recommended after resection with primary anastomosis, however its interest has never been studied. The aim of this trial is to define the role of systematic PS after resection and primary anastomosis for peritonitis Hinchey III and IV due to perforated diverticulitis.

**Methods/design:**

This DIVERTI 2 trial is a multicenter, randomized, controlled, superiority trial comparing resection and primary anastomosis with (control group) or without (experimental group) PS in patients with peritonitis Hinchey III and IV due to perforated diverticulitis.

Primary endpoint is the overall 1 year morbidity according to the Clavien–Dindo classification of surgical complications. All complications occurring during hospitalization will be collected. Late complications occurring after hospitalization will be collected during follow-up.

In order to obtain 80% power for a difference given by respective main probabilities of 67% and 47% in the protective stoma and no protective stoma groups respectively, with a two-sided type I error of 5%, 96 patients will have to be included in each group, hence 192 patients overall. Expecting a 5% rate of patients not assessable for the primary end point (lost to follow-up), 204 patients will be enrolled.

Secondary endpoints are postoperative mortality, unplanned reinterventions, incisional surgical site infection (SSI), organ/space SSI, wound disruption, anastomotic leak, operating time, length of hospital stay, stoma at 1 year after initial surgery, quality of life, costs and quality-adjusted life years (QALYs).

**Discussion:**

The DIVERTI 2 trial is a prospective, multicenter, randomized, study to define the best strategy between PS and no PS in resection and primary anastomosis for patients presenting with peritonitis due to perforated diverticulitis.

**Trial registration:**

ClinicalTrial.gov: NCT04604730 date of registration October 27, 2020. https://clinicaltrials.gov/ct2/show/NCT04604730?recrs=a&cond=Diverticulitis&draw=2&rank=12.

## Background

Up to 35% of patients treated for acute diverticulitis have a complicated disease such as purulent or faecal peritonitis (Hinchey III or IV) [1, 2]. However, the optimal strategy for the surgical treatment of peritonitis remains a subject of debate.

Traditionally, patients with peritonitis Hinchey III and IV due to perforated diverticulitis were treated with Hartmann’s procedure. In the past decade, resection and primary anastomosis have gained popularity over Hartmann’s procedure and recent guidelines recommend Hartmann's procedure in two situations only: critically ill patients and in selected patients with multiple comorbidity (at high risk of complications) [3, 4].

Although defunctionning stomas are recommended after resection with primary anastomosis, its interest has never been studied.

Protective stoma can effectively reduce the consequences of anastomotic leakage on clinical course but it also has its own morbidity. Theses complications cannot be neglected, and have been highlighted in several studies in rectal cancer; in addition its reversal requires a second operation which has been associated with more than 20% of morbidity [5–9].

Mengual-Ballester et al. reported a 45.9% incidence of ileostomy-related complications, including diarrhea, surgical wound infection, intestinal obstruction, rectorrhagia, enterocutaneous fistula, and anastomotic leakage [10].

Moreover, a protective stoma involves specific costs and can altere patient’s self-perception [11].

In the randomized trial “Diverti” [12] comparing Hartmann’s procedure vs resection and primary anastomosis, 15 of the 50 patients in the primary-anastomosis group were not protected. In this subgroup, mortality related to the operation was nil, and morbidity was only 26% (n = 4). In addition, severe complications (Clavien–Dindo III–IV) and global morbidity were significantly lower in the group of patients without protective stoma (0% vs. 23%, p = 0.042 and 27% vs. 67%, p = 0.01, respectively).

In the Ladies trial [13], patients operated with (n = 40) and without a protective stoma (n = 17) were also compared. No difference in overall morbidity (23.5% vs 45%, p = 0.15) and mortality (0 vs 7.5%, p = 0.55) was objectified, but patients without ileostomy had a significantly shorter median length of stay (7 vs. 11, p = 0.01).

These studies certainly have “selection biases”. Patients operated without protective stoma may have been “low risk” patients. However, the results appear very favorable.

Large scale studies do not exist and there is still debate as to whether or not defunctionning stoma should be performed routinely or should be avoided when possible [14].

Prospective randomized trials are needed to define the best strategy between PS and no PS in resection and primary anastomosis for patients presenting with peritonitis due to perforated diverticulitis.

## Methods

### Study objectives and design

**Fig. 1:**
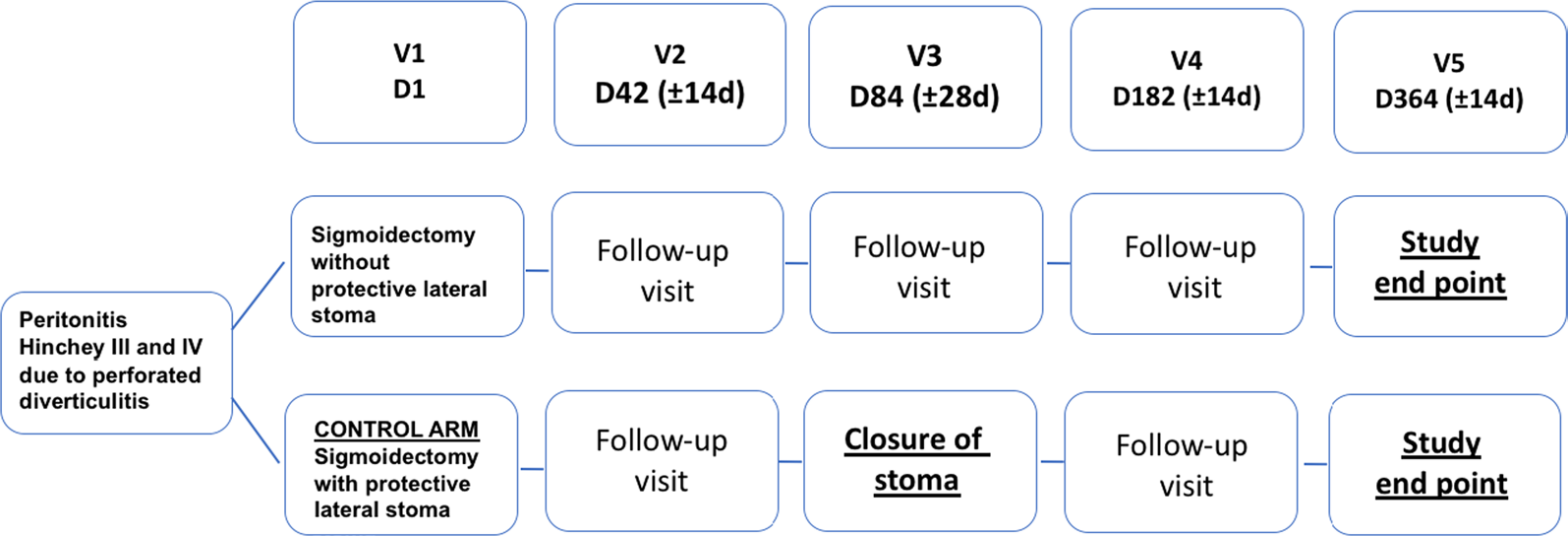
Study design of DIVERTI 2

This DIVERTI 2 trial is a multicenter, randomized, controlled, superiority trial comparing resection and primary anastomosis with (control group) or without (experimental group) PS in patients with peritonitis Hinchey III and IV due to perforated diverticulitis. Figure 1 summarize the design of the study.

The recruitment will last 36 months the duration of the study will be 48 months. This study is supported by a grant from the French Ministry of Health (PHRC-19-0229).

### Endpoints

The primary endpoint is the 1 year overall morbidity, i.e., the number of patients who develop at least one complication (medical or surgical) at the 12-month follow-up.

All complications occurring during hospitalization will be collected. Late complications occurring after hospitalization will be collected during follow-up.

A 12-month endpoint was chosen because 50% of patients will require stoma closure 3–4 months after emergency surgery. In addition, some complications related to the realization of a protective stoma can occurred in the long-term (incisional hernia for example).

Secondary endpoints are the 90 days mortality; number of re-admissions and re-operations (other than for stoma closure); Incisional Surgical Site Infections (SSIs); Organ/space SSIs; Wound disruptions; Anastomotic leaks; Operating time; Intensive Care Unit (ICU) length of stay; total duration of hospital stay; number of patients with a stoma at 12 months after initial surgery; quality of life (QoL) assessed by the Short Form Health Survey (SF-36) [15], the Gastrointestinal Quality of Life Index (GIQLI) [16] and the EuroQol Five-levels (EQ-5D-5 L) (i.e., utility values for health states) [17] at baseline 6, 12, 26 and 52 weeks after primary surgery. A 12-month cost-utility analysis, will be also performed. For patients benefiting from a restoration of continuity, the analyses will be performed on the cumulative data of the two interventions.

### Sample size

The calculation of the sample size is based on the results of DIVERTI trial [12], which evaluated the overall morbidity after protective stoma. The rate of patients with complications after protective stoma at 1 year was 67%.

We assume that the percentage of patients with complications in the protective stoma group of our study will be comparable to that of DIVERTI trial and we have based our calculation of the sample size on this information.

The sample size has been calculated with respect to Pearson’s chi-square test at two-sided 0.05 level to compare the proportion of patients with complications 12 months postoperatively between the two groups (protective stoma vs. no protective stoma).

In order to obtain 80% power for a difference given by respective main probabilities of 67% and 47% in the protective stoma and no protective stoma groups respectively, with a two-sided type I error of 5%, 96 patients will have to be included in each group, hence 192 patients overall.

Expecting a 5% rate of patients not assessable for the primary end point (lost to follow-up), 204 patients will be enrolled.

### Study population

Table 1 summarizes the inclusion and non-inclusion criteria.

**Table 1:** Inclusion and exclusion criteria

Inclusion criteria	Exclusion criteria
∙ Age ≥ 18 years ∙ Patients operated for purulent or fecal peritonitis (Hinchey stage III and IV) secondary to perforated diverticulitis of the left colon and treated by resection with primary anastomosis ∙ Patient having read and understood the information letter and signed the Informed Consent Form ∙ Patient able to comply with the study protocol, in the investigator’s judgment ∙ Patient affiliated with, or beneficiary of a social security (health insurance) category	∙ Physical states that prevent patient participation (e.g. septic shock or multivisceral failure) ∙ Steroid treatment > 20 mg daily ∙ Prior pelvic irradiation ∙ Immunocompromised status ∙ American Society of Anesthesiologists grade IV ∙ Peritonitis secondary to perforated diverticulitis of the right colon ∙ Patient is a pregnant or breastfeeding (lactating) woman or intending to become pregnant during the study ∙ Person deprived of liberty by administrative or judicial decision or placed under judicial protection (guardianship or supervision)

Inclusion criteria are patients older than 18 years with purulent or fecal peritonitis (Hinchey stage III and IV) secondary to perforated diverticulitis and able to provide informed consent.

Before enrollment, all patients have clinical assessment and CT scan and are informed about the study. The diagnosis is confirmed during surgery. Exclusion criteria are physical states that prevent patient’s participation (e.g. septic shock or multivisceral failure) and failure to provide consent.

### Participating centers

Twenty French public academic and non-academic hospitals will ensure recruitment.

A convention with the Direction of Research and Clinical Innovation of Rouen University Hospital, have been signed by all participating centers for ethical approval before beginning patient inclusion.

### Ethics

This study is conducted in accordance with the principles of the Declaration of Helsinki and ‘good clinical practice’ guidelines.

This study was submitted to the national drug agency (ANSM) and to the ethics committee “Comité de Protection des Personnes (CPP) Sud-Ouest et Outre-Mer II”, a national Institutional Review Board which belongs to the “Agence Regionale de Sante Occitanie”, on September 10th, 2020 (File number: 2-20-070 id8921); it was approved after minor corrections. Prior to randomization, written informed consent will be obtained from all patients. A new CPP assessment must be obtained for all protocol changes.

### Randomization

The investigator verify the eligibility of the patient with respect to the inclusion and non-inclusion criteria. After the delivery of oral information regarding the study, written consent is collected (day of inclusion). Both strategies are explained to the patients before enrollment in the study, along with their pros and cons. Randomization is performed just before surgery, by an online randomization system. Participating surgeons are allowed to login to the secured DIVERTI 2-trial website, after filling out the randomization form immediate response with randomization number and type of operation is obtained. The randomization list has been established using random number tables with a 1:1 ratio by a statistician before the initiation of the study. Randomization is stratified by center.

### Intervention

The patients are selected from the emergency department of each center. The diagnosis is established by the surgeon investigator on clinical data and imagery.

All consecutive patients with generalized peritonitis due to perforated diverticulitis are routinely proposed inclusion in the study. The decision of a possible inclusion is made after surgeon-anesthesiologist consultation. It’s the same if the patient’s condition changes peroperatively (infarction for example), a new surgeon-anesthesiologist consultation may need to modify the attitude initially adopted to adapt to the new clinical state of the patient.

The diagnosis of the non-tumor nature of the perforation is based on probability arguments and intraoperative findings. It isn’t be possible to obtain histological certainty of benign lesions before the first therapeutic act. Proof of diverticular perforation is required in each patient file at the end of the initial hospitalization.

Sigmoidectomy is performed through a midline laparotomy or laparoscopically, according to the standard technique, with lateral to medial mobilization of the left colon and mobilization of splenic flexure [12]. Vascular ligations are performed close to the intestine. The rectosigmoid junction is exposed and transected with a stapler. Proximal section is performed on a healthy colonic segment. The anastomosis is performed on well-vascularized digestive segments, without tension, according to the habits of the surgeon investigator (mechanical or manual anastomosis; end to end or side to end). After instillation of saline into the pelvis, the anastomosis is tested by air insufflation through the rectum. Decisions to clean the colon intraoperatively, to place a drain, and to perform ileostomy or colostomy (control arm) are left to the discretion of the surgeon.

In the control arm, stoma closure is performed at least 3 months after the first operation and after performing a barium enema to check for the absence of fistula or stenosis at the level of the anastomosis. Surgery is performed with a trephine incision, but the type of anastomosis (whether end-to-end or side-to-side, hand-sewn or stapled) is not restricted.

All patients are examined at 6, 12, and 26 weeks after the initial surgery, in the surgical department where they were operated; a final study visit will be carried out 12 months (evaluation of primary endpoint) after surgery.

The parameters explored at medical examinations are: occurrence of complications and quality of life assessment.

### Statistical analysis

Primary and secondary endpoints will be analyzed on an intention to treat basis. Data will be described overall and by randomized intervention group (protective stoma or no protective stoma) using standard parameters, i.e., mean, median, standard deviation, range and interquartile range for quantitative variables, and frequencies and percentages for categorical variables.

The main outcome being dichotomous, i.e., complication after surgery (yes/no), Pearson’s chi-square test or Fisher’s exact test will be used as appropriate to compare the two intervention arms 12 months postoperatively. Point and (exact if necessary) 95% confidence interval estimates of the proportions of complications in each arm will be calculated.

Use of the unadjusted test will be complemented by an adjusted comparison using the logistic regression model in order to adjust for center and possible prognostic characteristics.

The same general approach as outlined above will be used for secondary dichotomous outcomes (e.g., reinterventions, wound disruption, anastomotic leak). Regarding quantitative outcomes (e.g., operating time, length of postoperative hospital stay), comparison between the two intervention arms will be performed using Student’s test (or Mann–Whitney’s non parametric test as appropriate).

Quality of life scores will be described at each visit and compared between intervention groups at baseline and longitudinally during the study period using mixed linear models for repeated measures.

The overall survival at 3 months after surgery will be estimated using Kaplan–Meier survival curves and comparisons will rely on the logrank test.

The usual two-sided 0.05 level will be used for intervention comparisons.

### Economic assessment

The economic evaluation will be done over 12 months.

The result will be the quality-adjusted life year (QALY), derived from the l’EQ5D-5L questionnaire, as recommended by the French National Authority for health (HAS) [18].

#### Cost estimation

Direct medical costs will be calculated for each patient regarding the Diagnosis Related Group (Groupe Homogène de Séjour in French). Additional costs (resuscitation, intensive care, etc.) and non-reimbursed molecules will be considered in addition to the hospital stay. We will also estimate the direct costs related to complications of hospitalization. Nursing care and stoma replacement costs will also be estimated for stoma patients.

Indirect costs like work interruption will be estimated regarding the number of days not worked. A daily allowance will be estimated. Transports costs will include transport type, frequency and distance calculated according to the national health insurance rate in France.

Other costs will not be measured, but will be indirectly included in the Qaly measurement.

All costs will be calculated and presented in Euros (€) regarding current national health insurance rates in France.

#### Utility measure

QALY will be derived from the EQ5D-5L questionnaire. The EQ-5D-5Luestionnaire will be filled at 6, 12, 26 and 52 weeks after primary surgery. For patients benefiting from a continuity restoration, analyses will be performed on the cumulative data of the two interventions.

To avoid overestimating or underestimating patients’ utility, it will be estimated that this utility evolves in a linear way taking into account the time between each visit for each patient in the two groups.

Therefore, QALYs will be calculated as the utility × time interval between measures.

#### Medico-economic analysis

##### Cost-utility analysis

A cost and utility description of each group will be presented. Utility calculus will be presented in number of Qalys generated by each strategy. Mean and standard deviation with the 95% confidence interval will be estimated for each group.

##### Incremental cost-utility ratio (ICER) calculation and analysis

The incremental cost-utility ratio will be calculated at 12 months for the intervention group (strategy 1) versus the control group (strategy 2) with the mean costs and utility regarding each strategy. The incremental cost-utility ratio will be presented in additional cost per year of life saved (one QALY equates to 1 year in perfect health).

Sampling uncertainty around the cost estimates will be investigated using nonparametric bootstrapping. Re-sampling cost and utility pairs from the original sample will be used to generate 1000 replicates of mean differences in cost and utility. The resulting scatter plot will be used to derive cost-effectiveness acceptability curves (CEAC) by calculating the proportion of ICER replicates which would be considered cost-effective at various thresholds of willingness-to-pay (WTP) for an additional QALY.

### Data collection and monitoring

Data of each patient are collected via an electronic Case Report Form (eCRF) (base line, perioperative and long-term data), and all eCRFs are gathered by the coordinating center, Rouen University hospital.

After the initial surgery, all patients will be examined at 6, 12, and 26 weeks, in the surgical department where they were operated; a final study visit will be carried out 12 months (evaluation of primary endpoint) after surgery. The parameters explored at medical examinations are: occurrence of complications and quality of life assessment.

The entry of data are performed using Ennov Clinical® software (Ennov Group, 251 rue du Faubourg Saint Martin in Paris 75010—France). The database will be analyzed by the Biostatistics Unit of the University Hospital of Rouen.

During study conduct, the sponsor will conduct periodic monitoring visits to ensure that the protocol and Good Clinical Practices (GCPs) are being followed. The monitors may review source documents to confirm that the data recorded on CRFs is accurate.

This trial is registered on clinicaltrials.gov. The status is in progress: the first inclusion was June 15th, 2021.

## Discussion

This trial focuses on a surgical procedure for the treatment of diverticulitis peritonitis, one of the most commonly diagnosed surgical emergencies world-wide.

Although the Hartmann procedure was longtime considered as the gold standard for emergency surgery in diverticulitis, there is now a lot of data reporting significantly improved morbidity and mortality rates following resection and primary anastomosis. However the role of proximal diversion under these circumstances remains unclear. At present, there is no evidence-based medicine concerning the role of the protective stoma; current practice being essentially guided by old habits.

In fact, surgical removal of the pathologic process and control of sepsis remains the cornerstone of treatment for patients with perforated diverticulitis. Current surgical efforts should be to minimize the burden of operation with avoidance of creating a stoma and improving cosmesis without compromising the safety and efficacy of the procedure.

Obviously, defunctioning stoma reduces symptomatic anastomotic leakage after anterior resection of the rectum and is therefore recommended in rectal cancer resection with low anastomosis [19].

However, population of patients presenting with diverticulitis is different from that with rectal cancer: patients don't have a history of pelvic radiotherapy and the anastomosis is high. Resection and primary anastomosis without protective stoma is a recent and interesting approach in the management of diverticulitis peritonitis. However, this important topic has never been assessed in a randomized multicenter manner.

The low incidence of complications found in studies reporting this approach leads us to believe that protective stoma formation is not necessary in cases without obvious risk factors and when the general principles of a good anastomosis are followed. Our belief that stomas should be performed only in selected cases based on fact that stomy is not free of complications. Even more, if the surgeon feels that the anastomosis has major problems, conversion to terminal colostomy (Hartmann's operation) could be the best solution for the patient.

The DIVERTI 2 trial aims to test the hypothesis that sigmoidectomy with primary anastomosis without protective lateral stoma provides significant benefits in terms of reduced postoperative morbidity compared to conventional sigmoidectomy with primary anastomosis with a protective lateral stoma for peritonitis Hinchey III and IV due to perforated diverticulitis.

We believe that sigmoidectomy without protective lateral stoma can be a treatment option, since this technique allows preservation of normal intestinal continuity avoiding a protective stoma.

These patients may benefit from a lower rate of postoperative cumulative morbidity. In addition, impairment of quality of life in stoma carriers will be avoided without affecting functional outcome.

According to a lower rate of postoperative morbidity and the fact that closure of the stoma following surgery is not necessary, a reduction in direct costs (hospital stays and hospital admissions) may be expected. The introduction of sigmoidectomy without protective lateral stomy may likely be adopted in the future on a larger scale.

The DIVERTI 2 trial would be the first to study the role of protective stoma in patients with peritonitis Hinchey III and IV due to perforated diverticulitis who are candidates for sigmoidectomy with primary anastomosis.

It would also be the first study to evaluate the costs of protective stoma, and its impact on patient QoL.

## Data Availability

The study protocol is available on the website clinicaltrials.gov at the URL: https://clinicaltrials.gov/ct2/show/NCT04604730?recrs=a&cond=Diverticulitis&draw=2&rank=12. All data regarding ethical aspects are available at the Unité de Recherche Clinique—CHU Charles Nicolle. The materials that will be generated in this study will be available on request from the corresponding author.

## Abbreviations

ANSM: National Drug Agency
ASA: American Society of Anesthesiology
CPP: People Protection Committee
CT: Computed Tomography
DS: Defunctionning stoma
eCRF: Electronic Case Report Form
EQ-5D-5L: EuroQol Five-levels
GCPs: Good Clinical Practices
HAS: French High Health Authority
ICUR: Incremental cost-utility ratio
IPD: Individual Participant-level Data
LOS: Length of stay
PHRC: National Clinical Research Program
QALY: Quality Adjusted Life Year
QoL: Quality of life
SSI: Surgical Site Infections
